# Twilight Sleep

**Published:** 1915-01

**Authors:** 


**Twilight Sleep.** A symposium in the ''Medical Times,'' December, 1914, by 10 authorities, gives the general impression that the method has considerable value, though not adapted to general practice nor justifiable as a routine.

